# Predicting honest behavior based on Eysenck personality traits and gender: an explainable machine learning study using SHAP analysis

**DOI:** 10.3389/fpsyg.2025.1525606

**Published:** 2025-04-09

**Authors:** Yu Meng, Zili Peng, Zhitong Zhang, Qiaolin Chen, Hanxi Huang, Yihan Chen, Mengqian Zhao

**Affiliations:** College of Flight Technology, Civil Aviation Flight University of China, Deyang, China

**Keywords:** honest behavior, Eysenck personality, gender differences, machine learning, SHAP analysis

## Abstract

**Introduction:**

This study bridges a critical gap in aviation safety research by examining how Eysenck personality traits (Neuroticism, Psychoticism, Extraversion) and gender predict dishonest behavior in high-risk aviation contexts. While prior studies have focused on the Big Five and HEXACO models in ethical decision-making, empirical applications of the Eysenck framework to honesty prediction remain scarce-particularly in aviation, where dishonest acts (e.g., underreporting safety incidents) carry severe public safety consequences.

**Methods:**

We collected behavioral data from 102 flight and air traffic control cadets using a coin-toss task. Explainable machine learning (XGBoost) was employed to model nonlinear relationships between personality, gender, and honesty. Model performance was evaluated via AUC, with SHAP analysis identifying key predictors.

**Results:**

XGBoost achieved superior predictive accuracy (AUC = 0.802). SHAP analysis revealed: (1) neuroticism as the strongest predictor of dishonesty; (2) significant gender differences (higher dishonesty rates in males); and (3) threshold effects for Psychoticism and Extraversion.

**Discussion:**

This work makes three key contributions: (1) first systematic application of the Eysenck model to aviation honesty prediction; (2) identification of gender as a critical moderating variable; and (3) development of a SHAP-driven interpretable framework that connects machine learning outputs with psychological theory. Practically, these findings enable data-driven screening of cadets' honesty tendencies during recruitment, facilitating targeted interventions for safer aviation operations.

## Introduction

### Background

Generally, honest behavior is a type of moral virtue in social life, influencing personal live, while having a large impact on society. For example, research has found that dishonest behavior reduces individuals' emotional empathy, thereby impacting their social interactions and ethical behavior (Lee et al., 2019). As a result, businesses incur additional economic burdens to monitor and manage employees' dishonest practices. Consumers, driven by distrust, may pay higher prices to ensure the authenticity of their purchases, leading to psychological strain. Similarly, governments allocate more resources to combat dishonest behavior, resulting in increased public expenditure and strained law enforcement resources (Gomes et al., 2022). Therefore, honesty is of paramount importance across various societal domains.

In the aviation sector, honesty is more important since such situations involve direct implications for public safety. In the field of aviation psychology, human factors are a critical element in the causation of aviation accidents and decision-making processes (Koglbauer, 2018). Researchers investigating factors influencing flight safety in Chinese civil aviation through HFACS-based event analysis have found that human factors significantly impact flight safety. Specifically, communication and coordination among team members, as well as organizational culture and atmosphere, play a crucial role in ensuring safety during flights (Zhang et al., 2007). However, dishonest behavior is a prevalent issue in the aviation sector. Attitudes toward organizational culture and flawed cultural practices may discourage employees from voluntarily reporting safety-related information (McMurtrie and Molesworth, 2018). Additionally, fear of damaging colleague relationships or personal interests can lead flight crew members to remain silent when faced with issues or critical events that may compromise flight safety (Ceken and Unsal, 2023). A study exploring pilots' voluntary reporting behavior in aviation safety management revealed that 53% of Australian pilots and 33% of European pilots either fail to report or partially report such incidents (McMurtrie, 2020).

The issue of dishonest behavior in aviation has been highlighted in several aviation incidents, underscoring its dangers. For instance, the co-pilot of Germanwings Flight 9,525 suffered from mental illness and concealed his medical history, intentionally crashed the plane, resulting in the deaths of 150 people. Subsequent investigations into pilots' mental health revealed that the prevalence of depression among pilots ranges from 1.9% to 12.6% (Pasha and Stokes, 2018). In another study involving 61 pilots from aviation accidents where selective serotonin reuptake inhibitor (SSRI) antidepressant residues were detected, it was found that most pilots did not report their use of antidepressants, despite SSRIs not being approved for use by U.S. civil aviation personnel. Furthermore, 88% of the pilots chose not to disclose their psychological conditions (Sen et al., 2007). These findings emphasize the critical importance of addressing dishonest behavior in aviation to ensure safety and prevent catastrophic outcomes. According to the U.S. Department of Justice (2021), one former chief Boeing 737 MAX technical pilot was indicted for fraud by providing false information during the evaluation of the aircraft that hugely compromised flight safety oversight. A similar case happened when Cathay Pacific banned its trainee pilots from solo flights after their attempts to hide incidents (Zaobao, 2024). More disturbingly, it came to light that over 30% of the active pilots of Pakistan had fake flying licenses, again showing just how pervasive and hazardous scent of fraud was in aviation (Nan, 2020). These cases illustrate that the personal character of professionals in aviation is not an issue but rather a part of public safety and the long-term development of the industry. Research indicates that human factors are key variables in predicting the fatality of aviation accidents, and they significantly influence accident outcomes (Lázaro et al., 2024). However, within the field of aviation safety research, empirical studies predicting dishonest behaviors through the human factors framework remain critically scarce. This research gap significantly impedes the further refinement of aviation safety management systems. Therefore, continuous efforts are still required to predict and promote honest behavior among aviation personnel.

Current research on predicting dishonest behavior typically focuses on neuroscience, social contexts, and individual characteristics. On one hand, in the field of neuroscience, studies have shown that the neural representations of honest and dishonest behaviors primarily occur in the posterior cingulate cortex (PCC), bilateral dorsolateral prefrontal cortex (DLPFC), and left intraparietal sulcus (IPS; Bellucci et al., 2019). Furthermore, fMRI studies have revealed that the hypothesis-driven network plays a significant role in predicting dishonesty in multitasking behaviors. This network includes midline self-referential brain regions (medial prefrontal cortex, posterior cingulate cortex, and anterior cingulate cortex), anterior insula, and striatum (Guo and Yin, 2024). Additionally, another study on cheating behavior found that functional connectivity between the dorsolateral prefrontal cortex (dlPFC), medial prefrontal cortex (MPFC), inferior frontal gyrus (IFG), supplementary motor area (SMA), temporal pole, posterior cingulate cortex (PCC), and caudate nucleus is crucial for predicting honest behavior. These brain regions are associated with self-referential thinking, cognitive control, and reward processing (Speer et al., 2020). On the other hand, in research on social contexts for predicting honest behavior, data collected through questionnaires, behavioral observations, and self-reports revealed that actual feelings of pleasure and social connectedness were significantly higher than expected in honest conditions. Compared to other control conditions, honesty fostered greater social connection and pleasure (Levine and Cohen, 2018). Sulitzeanu-Kenan et al. (2022) analyzed data from Cohn et al.'s “civic honesty” field experiments conducted in 40 countries (Cohn et al., 2019) and found that public sector honesty is a strong predictor of regional corruption levels. Higher public sector honesty correlates with lower corruption levels. Moreover, experimental paradigms such as the Trust Game (TG) and Take Advice Game (TAG) demonstrated that trust levels can predict dishonest behavior dishonest behavior, as individuals are more likely to trust those who share truthful information (Bellucci and Park, 2020). Beyond neuroscience and social contexts, individual characteristics are also a significant factor in predicting dishonest behavior. Our research focuses on two key aspects of individual traits: personality and gender.

In the course of research development and application of various personality models, it was also unwrapped how individual personality traits attributed to the Big Five Personality Model, the HEXACO Model, and Eysenck's Personality Model relate to dishonest intent and behavior. The Big Five personality model is the most widely accepted framework for describing personality structure, encompassing five dimensions: extraversion, agreeableness, conscientiousness, openness, and neuroticism. Extraversion refers to an individual's level of sociability and confidence in social settings. Agreeableness reflects an individual's tendency to be friendly and cooperative. Conscientiousness involves an individual's organizational skills and reliability. Openness indicates an individual's receptiveness to new experiences. Neuroticism describes an individual's propensity to experience negative emotions (Abood, 2019). It was, in fact confirmed by He and Gong (2010) that the Big Five model was closely related to honesty, while Malesky et al. (2022) underpinned openness as key in fostering ethical behavior. Furthermore, Kokkinos et al. (2023) reported emotional stability and agreeableness as significant predictors of dishonest behavior. However, the argument by Marcus et al. (2007) is that the Big Five model cannot fully account for the personality-honesty relationship, hence the need to introduce the HEXACO model-including the “honesty-humility” dimension-to gain a better understanding of the role of personality in influencing ethical behavior. The Honesty-Humility (H) dimension in the HEXACO personality model is typically defined as an individual's tendency to exhibit sincerity, fairness, and a lack of interest in material wealth and social status in interpersonal interactions. In predicting materialism, social adroitness, criminal behavior, and unethical decision-making, the HEXACO model demonstrates higher predictive validity (Ashton et al., 2014). Supporting this approach, Scigała et al. (2019) describe a significant negative relation of dishonest behavior in cooperative contexts with the honesty-humility trait according to the HEXACO model; if anything, this suggests that people higher on Honesty-Humility are less likely to engage in cheating. This finding was subsequently supported by other research which showed that the ones low in Honesty-Humility will more often choose an environment that would allow them to continue their dishonest actions (Houdek et al., 2021).

Another perspective on how personality influences dishonest behavior is provided by Eysenck's personality model. The Eysenck personality model comprises three primary dimensions: Neuroticism-Stability, Introversion-Extraversion, and Psychoticism. The Neuroticism-Stability dimension reflects differences in individuals' emotional stability and the intensity of their emotional responses. The Introversion-Extraversion dimension describes variations in individuals' social behaviors and energy acquisition patterns. The Psychoticism dimension captures differences in impulsivity, callousness, and unconventional thinking and behavior (Eysenck, 2016). Unlike the self-report questionnaires used by other researchers at the time, Eysenck's data were derived from intake rating scales completed by psychiatrists, including actual behaviors or behavioral ratings such as age, employment status, type of work, psychological abnormalities in parents or siblings, and alcohol consumption levels. These scales not only describe how individuals perceive themselves or how others perceive them but are also based on individuals' actual behaviors (Revelle, 2016). However, the Eysenck scales appear to have limitations in cross-cultural applicability (Yang et al., 2023), and the neglect of complex cognitive processes (Matthews, 2016). The relationship between personality and dishonest behavior differs from the two traditional personality trait models mentioned above. A meta-analysis by Lee et al. (2019) entails that psychopathy and impulsivity are strongly related to dishonest acts, lots of which may imply emotional and behavioral control in dishonest behavior. Neuroticism and psychoticism, as proposed by Eysenck, reveal emotional and behavioral control, respectively. Studies have shown that a higher degree of neuroticism scores leads to more dishonest behavior, and the high psychoticism scores are also related to higher levels of dishonest behavior. It might be because of aggressive reactions in situations (Barnes and Malamuth, 1998) given or because of the externalizing mechanisms like lying to cope with the problems given (Dodaj et al., 2021). More dishonest behavior may also manifest in highly neurotic individuals because emotional instability or arousal taking place in response to negative emotions leads them to commit moral offenses as a way of reducing anxiety (Peters et al., 2020). In contrast to neuroticism and psychoticism, Eysenck's extraversion factor tends to affect dishonest behavior in a different direction. The reason for this may be that some of these unethical behaviors (Xiong et al., 2022) are not significantly related to extraversion because individuals with low extraversion tend to lose interest in tasks or use lower connectedness to the task at hand (Padrell et al., 2020). Though Eysenck's personality theory is one of the foundational models, much research applying his theory specifically to dishonest behavior has been quite limited, and not enough systematic and large-scale investigation has tried to explore the direct relationship between these traits and dishonest behavior.

The above discussion clarifies research on different personality models and dishonest behavior, revealing that each model has distinct advantages in predicting dishonest behavior but also certain limitations. For the Eysenck personality model, although it is one of the classic personality frameworks, its application in dishonest behavior research is limited, with few large-scale studies directly exploring the correlation between its traits and dishonest behavior. However, existing evidence in the literature suggests that this model may have predictive capabilities in areas such as emotional regulation and impulse control (Barnes and Malamuth, 1998; Peters et al., 2020). The Honesty-Humility dimension in the HEXACO model directly measures traits such as honesty, fairness, and the suppression of material desires, significantly enhancing the predictive validity for dishonest behavior. However, there may be an overemphasis on social desirability in item phrasing, and the classification of adjectives may overlap with other dimensions, potentially affecting the independence of the Honesty-Humility trait. Additionally, the HEXACO model focuses more on the shared variance among these traits (Ashton and Lee, 2020; Gill and Berezina, 2019; Hodson et al., 2018; Howard and Van Zandt, 2020), whereas we are more inclined to study the impact of individual personality traits on pilot behavior. Although the Big Five personality model is a widely accepted framework for personality structure, it is more strongly correlated with positive aspects of personality. Its predictive ability for certain negative personality traits is limited when applied to high-risk occupational groups (Paleczek et al., 2018). Furthermore, the Big Five model may not encompass all aspects of personality, and since it influences behavior through complex interactions with the environment, it becomes challenging to fully predict or explain individual behavior in specific contexts (McCrae and Costa, 2021). Meanwhile, the 16PF model has not sufficiently explained the influence on dishonest behavior (Boyle et al., 2016; Marcus et al., 2007; Riggio et al., 1988). The strengths and weaknesses of personality models in the field of honest behavior research are compared in Table 1.

**Table 1:** Comparison of the advantages and disadvantages of personality models.

**Personality models**	**Advantages**	**Disadvantages**
The Big Five personality model	**Universality**: A widely accepted personality framework, facilitating cross-study comparisons (Abood, 2019). **Dimensional relevance**: Agreeableness and conscientiousness are positively correlated with **honest** behavior (Kokkinos et al., 2023), while openness promotes moral behavior (Malesky et al., 2022).	**Limited predictive power for high-risk contexts**: Insufficient predictive capability for negative personality traits (Paleczek et al., 2018). **High environmental dependency**: Behavior is influenced by complex environmental interactions, making it difficult to predict performance in specific situations (McCrae and Costa, 2021).
The HEXACO model	**Direct measurement of honesty traits**: The addition of the “Honesty-Humility (H)” dimension effectively predicts behaviors such as fraud and cheating (Ashton et al., 2014). **Cross-cultural applicability**: Demonstrates high face validity (Howard and Van Zandt, 2020).	**Risk of dimension overlap**: Adjectives describing **Honesty**-Humility may overlap with other dimensions, such as Agreeableness (Gill and Berezina, 2019). **Issue of shared variance**: The model focuses on the common variance among traits, making it difficult to isolate the impact of individual traits on behavior (Hodson et al., 2018).
The Eysenck personality model	**Emotion regulation mechanism**: Demonstrates predictive capability in the domains of emotion regulation and impulse control (Peters et al., 2020).	**Limited usage**: Primarily used in psychiatric contexts, limitations in cross-cultural applicability (Yang et al., 2023), the neglect of complex cognitive processes (Matthews, 2016). **Lack of research, significant gaps**: Rarely applied in studies on **dishonest** behavior, leaving substantial research gaps.

Given the strengths and limitations of the aforementioned personality models, this study selects the Eysenck personality model as a representative framework for examining the predictive capabilities of personality traits related to emotion regulation and impulse control in the context of dishonest behavior, thereby addressing the research gap in this area. Additionally, the choice of the Eysenck model aligns with the psychological assessment criteria used in the recruitment process for pilots in China's civil aviation industry. The training requirements for flight crew also emphasize psychological factors related to emotional stability and regulation (Civil Aviation Administration of China [CAAC], 2017). We predict that leveraging personality traits to predict honest behavior will extend the application of psychological assessments to the pilot screening phase.

Apart from personality traits, gender is another individual characteristic that consistently garners central attention in research on honesty-related behaviors. Numerous studies affirm that gender plays a pivotal role in shaping personal honesty, as men and women exhibit differences in moral attitudes, societal expectations imposed upon them, and decision-making tendencies (Eagly, 2013; Harenski et al., 2008). The primary drivers of these disparities encompass socialization processes, cultural contexts, and psychological influences.

From the perspective of socialization, men and women lead different social expectations during development. According to Social Role Theory, the social roles assigned to them dictate that men and women develop certain basic behavioral propensities. For example, men are usually socially encouraged to be competitive and independent, while for women, it is expected to be caring and cooperative (Eagly, 2013). These differences in social roles stem from the activation of distinct neural regions in males and females when confronted with dishonest behavior. For example, Research indicates that women rely more on neural regions associated with care and empathy (posterior cingulate cortex and anterior insula) during moral evaluations, leading to more emotionally driven moral decision-making compared to men (Harenski et al., 2008). This tendency makes women more inclined toward honesty in similar situations. On the other hand, men may be dishonest owing to expectations set by society of competition and success (Huang and Hung, 2013).

Moral Emotion Theory still postulates that women are more sensitive to these moral emotions of shame and guilt, because, therefore, in situations which involve dishonest behavior (Tangney et al., 2007), such women develop stronger moral constraints. Family and educational settings support the view that parents stress rule awareness and obedience more for girls than boys; in addition, boys are frequently taught to be more assertive and independent (Kennedy and Kray, 2022). This difference in socialization influences the honesty behaviors based on gender and might come along with striking contrasts in behaviors within given contexts. For example, in a setting where there are economic rewards for being dishonest, men show higher propensities for taking risks. In firms where monitoring mechanisms are weak, male-run businesses are most likely to present fraudulent reports (Gupta et al., 2020).

Male also tend to commit fraudulent behaviors across various situations, from academic settings all the way to business dealings and even into leadership, using deceit or actions of empty promises for personal gain (Kennedy and Kray, 2022). To that effect, such might be premised on men being more predisposed toward taking risks, since fraudulent behavior is usually considered a high-risk, high-reward strategy.

While women are generally more moral than men, there are some contexts in which women might cheat more. For instance, research has documented that offering a leadership role to women, particularly in team-based decision-making, can encourage dishonest behavior among women. This appears to occur because female leaders are at times swayed by the fraudulent preferences of teammates (Grosch et al., 2022). These findings illustrate how gender differences in honesty are formed not just through socialization and moral emotions but also through context and expectation of a certain role that is being played.

Since there are known gender differences in dishonest behavior, gender has been used as a significant predictor in this study.

Model-based classification methods were widely used for several earlier studies about personality and behavior prediction. For instance, several works employed the Finite Mixture Models (FMM) to conduct some analyses concerning estimated effects of personality traits on behavior (Rahm-Knigge et al., 2018). Model-based approaches bring an important theoretical framework that allows researchers to identify some behavioral patterns across different personality types in certain situations. While most are based on static models, fewer have utilized the fast developing machine learning algorithms of recent years, such as decision trees, random forests, support vector machines, and deep learning.

While machine learning methods are, of course, comparably advantageous to deal with data complexity and non-linear relationships, they enhance the accuracy and efficiency of prediction on big data. Algorithm applications such as neural networks and random forests in studies like Dunne et al. (2023) and Song (2022) have been competently documented to be particularly effective in predicting abnormal and routine behaviors at specific levels in complex dynamic environments. These machine learning algorithms will not only increase the accuracy of behavior prediction but can also integrate more kinds of data sources, such as sensor data (Kambham et al., 2018) and social media records (Zumma et al., 2022), for wider applications in research.

Besides behavior prediction, machine learning algorithms have also been in wide use for the analysis of individual characteristics, such as gender prediction. For example, Liu et al. (2021) proposed the method of gender prediction that integrates emotional characteristics of users for social media user profiling and identified the importance of gender in predicting social media behavior. For the gender prediction, Bijoy et al. (2022) applied different machine learning algorithms to peoples' preferences in choosing a mate, and through random forest, it attained an accuracy of 95.39%. Likewise, in analyzing students' online learning behavior, Yan and Au (2019) applied machine learning for academic performance prediction and investigated the moderating effects of gender and age on learning outcomes. These studies reflect significant development in using machine learning for the prediction of gender characteristics and show that gender information stands out as very important for predicting behavioral performance.

Applications of machine learning in this area have established the fact that personality traits indeed can be inferred from behavior: for instance, one work by Salima et al. (2023) offered successful prediction of Big Five personality traits based on eye-tracking data and another work by Naik et al. (2022) used deep learning for the analysis of online text and prediction of MBTI types. Works like these establish that extraction of underlying personality traits from behavioral data of language and physiological signals is possible. Meanwhile, the importance of the role of gender factors in behavior prediction is increasingly being recognized. Previous studies showed that the combination of gender and personality traits with some techniques of machine learning may result in increasingly more accurate models in forecasting individual behavior.

Personality prediction also used data from videos and social media. The neural network algorithms predict Big Five personality traits through video imagery, emotional analysis, and Kunte and Panicker (2019) use Naive Bayes among other machine learning algorithms to predict these personality traits from the text in social media. Smartphone sensor data also proved useful for personality prediction, with Kambham et al. (2018) developing the corresponding machine learning models. These studies illustrate the wide application of machine learning in personality studies.

This study aims to predict individuals' dishonest behavior in reward-driven scenarios using the Eysenck personality model (Neuroticism, Psychoticism, Extraversion) and gender characteristics, leveraging machine learning. It also explores the influence of personality traits and gender on dishonest behavior. Our research questions focus on two main aspects: Which and how do Eysenck personality traits (Neuroticism, Psychoticism, Extraversion) influence individuals' dishonest behavior in a coin-tossing task? Does gender independently exert a significant impact on dishonest behavior beyond personality traits? Our conceptual model posits that Neuroticism increases the likelihood of dishonest behavior, and gender differences manifest distinct patterns in dishonest behavior. Based on this, the hypotheses for the study are as follows: (1) Neuroticism increases the likelihood of dishonest behavior. (2) Psychoticism increases the likelihood of dishonest behavior. (3) Males are more likely to exhibit dishonest behavior than females. (4) Gender and personality traits jointly influence dishonest behavior.

## Methods

### Participant

**Figure 1:**
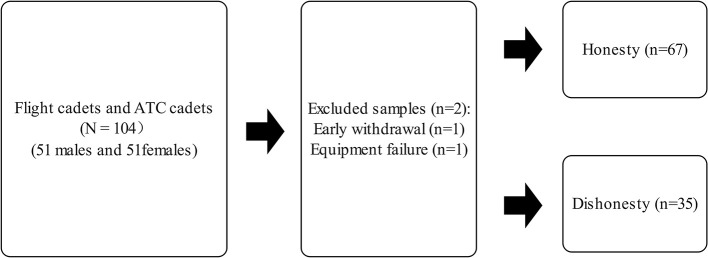
Flowchart of subject's selection. ATC, Air Traffic Control.

This study focuses on flight cadets and ATC cadets as research subjects, as these individuals will assume critical roles in the civil aviation sector in the future. Identifying their honest behavior holds significant value for personnel management and safety assurance in the aviation industry. Detecting honest behavior during their training phase can serve as a reference for screening honesty in high-risk positions later. To determine the sample size, an *F*-test was selected as the statistical method, with an effect size set at 0.15, statistical power at 0.8, and a significance level of 0.05. Using G-Power, the total required sample size was calculated to be 98. A total of 104 flight cadets and ATC cadets were randomly recruited from the Civil Aviation Flight University of China, with 102 participants ultimately included in the final cohort of the study. The selected flight cadets (mean age: 23.435 ± 0.779 years, mean flight h: 237.030 ± 5.239 h) had all passed the CAAC-mandated exams for single-engine and multi-engine private pilot licenses (PAE) and instrument rating exams (IRE). Their training aircraft included the C-172, SR-20, DA-42NG, and PA-44. The ATC cadets (mean age: 20.893 ± 0.493 years, mean simulated tower hours ≥ 72 h) had all passed the CAFUC-mandated exams for airport control, procedural control, and radar control. Their training simulated towers included airport control desktop CBT and a 120-degree airport control simulated tower. The participant screening process and results, including the Number of participants, are detailed in the Figure 1.

### Research design

Our study employs a mixed experimental design, with factors being personality traits (neuroticism, psychoticism, extraversion) and gender (male, female). Personality traits and gender serve as independent variables, while honesty behavior is the dependent variable. The experiment is designed to create an Opportunity condition, manipulate, and measure individual honesty levels through a variation of a “coin flip task,” a well-established experimental paradigm that effectively uncovers participants' honesty tendencies. The selection of this paradigm is theoretically grounded in three aspects: First, its probability distribution characteristics (binomial distribution) provide a statistical basis for detecting abnormal reporting, serving as an objective quantitative indicator of dishonest behavior. Second, the immediate economic incentive mechanism (a reward of 1 RMB for correct predictions) simulates real-world scenarios of temptation for personal gain, offering ecological validity similar to situations where aviation professionals might face opportunities for illicit profit. Third, research has shown that this task effectively activates the prefrontal control network (DLPFC/VLPFC), with its neural characteristics serving as biological markers for honest decision-making (Greene and Paxton, 2009).

### Experimental purpose

The study aims to determine if personality traits and gender influence participants' honesty responses in a reward-driven context, creating opportunities for dishonest behavior.

### Experimental environment

All experiments are conducted in a controlled, distraction-free laboratory setting, which contains only the necessary equipment and participants to minimize external disturbances and ensure data reliability.

### Experimental preparation

During recruitment, participants were informed that the study sought “individuals with supernatural abilities” targeting flight cadets and ATC cadets within the school who met the required operational experience. They were told the study aimed to test precognitive abilities and could enhance our understanding of the relationship between human cognition and paranormal phenomena. This approach was designed to conceal the true experimental purpose and avoid the influence of social desirability bias on the results. Upon arrival at the laboratory, participants were informed that the experiment aimed to precognitive abilities” (a cover story) and were asked to sign an informed consent form (10 min). Subsequently, they completed the Chinese version of the Eysenck Personality Questionnaire Short Scale (EPQ-RSC; Qian et al., 2000; 15 min), a demographic questionnaire including gender, age, living expenses, and region (10 min), and the Revised Paranormal Belief Scale (RPBS; Tobacyk, 2004; 10 min). The inclusion of the paranormal belief scale further masked the true purpose of the experiment. Participants were informed that the base payment for the experiment was 30 RMB, with 100 opportunities to make predictions. They would earn an additional 1 RMB for each “correct prediction,” with the final payment calculated as “Base 30 RMB + Number of correct predictions × 1 RMB.” Payments were settled and disbursed immediately after the experiment, with an average reward of 87.951 RMB.

### Experimental task

**Figure 2:**
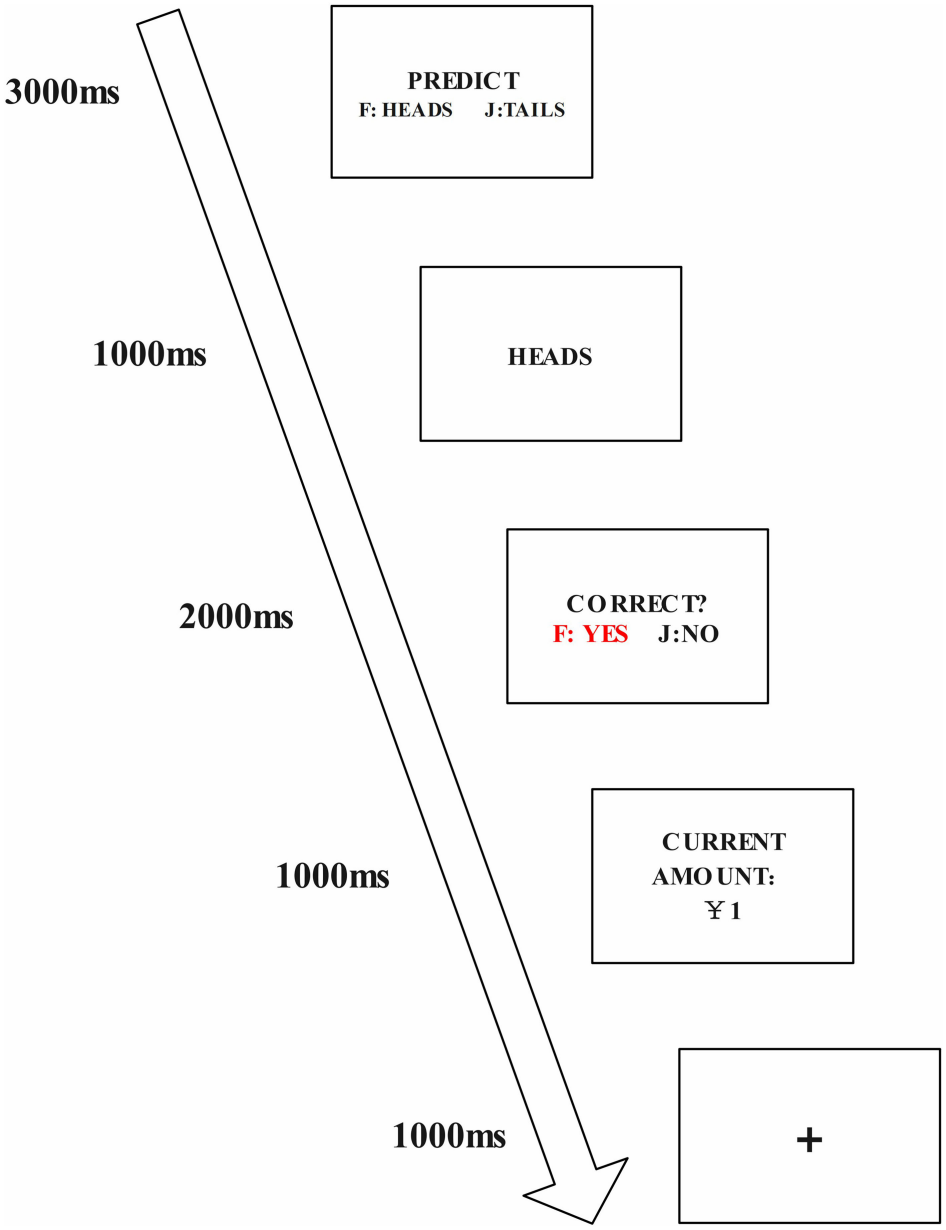
“Coin flip guessing” game flowchart. Reward: 1 RMB for correct guess.

The task, depicted in Figure 2, involved a “coin flip guessing” game where participants predicted the coin flip outcome before it was revealed and recorded their prediction.

The experimental task adopted the “coin toss prediction” paradigm, as illustrated in Figure 2. The experimental interface displayed instructions, and participants were explicitly informed that the experiment would consist of five screens. The first screen showed a black cross at the center for 1,000 ms to focus participants' attention. The second screen displayed a rotating one-yuan coin at the center, and participants were instructed to predict its orientation when it stopped spinning by pressing the F key for heads or the J key for tails. After the coin rotated for 3,000 ms, the screen automatically transitioned to the third interface. The third screen showed the coin's final orientation at the center for 1,000 ms, followed by an automatic transition to the fourth screen. The fourth screen asked participants whether their prediction in the first window was correct, with the F key indicating “correct” and the J key indicating “incorrect.” After 2,000 ms, the screen transitioned to the fifth interface. The fifth screen updated the cumulative reward based on the participant's choice in the fourth interface, adding 1 RMB each time the F key was pressed, and was displayed for 1,000 ms. Participants could start the experiment by pressing the spacebar, and the experiment consisted of 100 trials. The system randomly controlled the ratio of correct to incorrect guesses at 1:1.

### Measurement of dishonest behavior

The experiment established scenarios to measure dishonest behavior, focusing on the discrepancy between predicted and registered outcomes, particularly in scenarios where predictions were incorrect but registered as correct.

### Machine learning model and analysis

This study utilized various machine learning algorithms to determine if Eysenck personality traits, gender, and other demographic data could reliably predict individual honesty behavior. A comprehensive description of the model selection and training process follows.

### Feature selection

Features analyzed included Eysenck personality traits (neuroticism, psychoticism, extraversion), gender (male = 1, female = 2), living expense levels (classified by living expense tier, with tiers ranging from 1 to 9), and living regions (classified by city tier, with tiers ranging from 1 to 4).

### Model selection

The decision to employ machine learning algorithms rather than traditional static models in this study is based on the following reasons:

(1) Sensitivity to Research Questions: This study aims to explore the non-linear relationships and interaction effects between personality traits (Neuroticism, Psychoticism, Extraversion) and gender on honest behavior. Traditional linear models (e.g., Pearson correlation, linear regression) can only capture linear associations between variables, whereas real-world behavioral data often involve complex feature interactions and non-linear relationships (e.g., threshold effects, dynamic modulation). For example, our Pearson correlation analysis revealed that only gender was significantly correlated with honest behavior (*p* = 0.022), while the significance of Neuroticism was close to the threshold (*p* = 0.096), suggesting its influence may operate through non-linear pathways or interact with other variables. The “black box” nature of machine learning algorithms is often utilized to identify such complex patterns, enabling more sensitive detection of dynamic relationships between variables. However, this “black box” characteristic reduces interpretability, necessitating the use of SHAP analysis to enhance the explainability of machine learning models.
(2) Adaptability to High-Dimensional Feature Spaces: Although the feature dimensions in this study (six predictor variables) do not meet the “high-dimensional” standard, the relationship between personality traits and behavior often exhibits high heterogeneity (e.g., the impact of Neuroticism on honesty may vary by gender or living environment; McCrae and Costa, 2021). Traditional statistical models require pre-specification of interaction terms, whereas machine learning, through feature importance assessment and SHAP value analysis (Lundberg and Lee, 2017), can unsupervisedly capture potential interaction effects between variables.
(3) Compatibility with Interpretability Tools: Although traditional statistical models (e.g., logistic regression) offer parametric interpretability, their assumptions (e.g., linearity, independence) often do not hold in behavioral data. We chose machine learning algorithms combined with SHAP analysis, as this approach provides visual explanations of the model. It retains the predictive performance of machine learning while offering interpretability comparable to traditional models through visual methods (e.g., feature importance ranking, individual prediction attribution). Therefore, we opted to use machine learning algorithms in this study.

Selected algorithms comprised XGBoost, Particle Swarm Optimization Support Vector Machine (PSO_SVM), Random Forest, Naive Bayes, Logistic Regression, LightGBM, CatBoost, and Transformer. These models, known for their efficacy in classification tasks, are capable of managing high-dimensional and non-linear data. Each model presents unique advantages; for instance, XGBoost manages complex feature interactions well, Random Forest minimizes overfitting risks, Support Vector Machine excels in small sample classifications, Logistic Regression is suited for straightforward binary tasks, and CatBoost is optimized for categorical data.

### Data division and processing

The data was then divided into training and testing sets at a ratio of 80:20. Since the sample size is small, the five-fold cross-validation method was considered to avoid overfitting and ensure the stability of the model. The chosen ratio will promote this form of calculation. In the present study, the dishonest behavior was taken as positive, which is coded as 1, while the negative is coded as 0.

### Model evaluation and comparison

Since it was hard to know in advance which model configuration would work best, the Grid search was used in order to make a hyperparameter optimization. The hyperparameters of each model have been optimized in training, be it kernel functions in the case of the Support Vector Machine or the number of decision trees, which corresponds to Random Forest. The training strategy employed early stopping (early_stopping_rounds) and utilized five-fold cross-validation. For instance, the hyperparameters of the XGBoost model were configured as follows: learning rate (learning_rate) = 0.3, maximum tree depth (max_depth) = 6, the number of trees determined by early stopping (n_estimators = “early_stopping_rounds”), sample sampling rate (subsample) = 0.80, L2 regularization coefficient (reg_lambda) = 1, and the logarithmic loss metric (eval_metric = “logloss”) combined with L2 regularization to prevent overfitting. Other models, such as logistic regression, random forest, and SVM, adopted the default parameters from “scikit-learn.”

Various evaluation metrics such as the Area Under the Curve (AUC), 95% confidence interval, Youden's Index, specificity, sensitivity, *F*_1_ score, and accuracy. Additionally, Positive Predictive Value (PPV) and Negative Predictive Value (NPV) were used to demonstrate the proportions of true positives and true negatives in the classification results, thereby highlighting potential misclassification scenarios. Among stability, adaptability, and the highest sensitivity, XGBoost, as the best-performing model, was selected. The interpretability of the model was further enhanced using the SHAP (SHapley Additive exPlanations) method.

### Machine learning interpretability tool

However, interpretability of these models was made using the SHAP method, which is a unified approach of calculating the contribution and impact of each feature with high precision toward the final prediction. SHAP values explain whether the effect of each predictor variable is positive or negative for the target variable. Furthermore, each observation in the dataset can be explained through a specific set of SHAP values. Then, with respect to the dishonest behavior, the contribution analysis of different personality dimensions and gender was performed, ranking the importance of features in the model by SHAP.

## Results

### Descriptive statistics

Table 2 presents the descriptive statistical analysis of the continuous variables in the study, with a sample size of 102 (*N* = 102). The mean (*M*), standard deviation (*SD*), skewness, and kurtosis of each variable are as follows: The mean for Neuroticism is 18.59 (*SD* = 3.434), with a skewness of −0.287 and kurtosis of −1.035, indicating a slightly left-skewed and relatively flat distribution. The mean for Extraversion is 15.78 (*SD* = 2.680), with a skewness of 0.695 and kurtosis of −0.540, showing a right-skewed and slightly flat distribution. The mean for Psychoticism is 17.24 (*SD* = 1.401), with a skewness of −0.497 and kurtosis of 0.148, reflecting a slightly left-skewed distribution close to normal. The mean for urban classification is 3.21 (*SD* = 1.018), with a skewness of −0.771 and kurtosis of −0.934, indicating a left-skewed and flat distribution. The mean for living expenses is 4.01 (*SD* = 2.104), with a skewness of 0.787 and kurtosis of 0.178, showing a right-skewed and slightly peaked distribution. Overall, none of the variables exhibit extreme skewness or kurtosis, and the data distributions are reasonable, making them suitable for further analysis.

**Table 2:** Descriptive statistical analysis of continuous variables.

**Variable**	***N***	***M***	***SD***	**Skewness**	**Kurtosis**
Neuroticism	102	18.59	3.434	−0.287	−1.035
Extraversion	102	15.78	2.680	0.695	−0.540
Psychoticism	102	17.24	1.401	−0.497	0.148
Living regions	102	3.21	1.018	−0.771	−0.934
Living expense levels	102	4.01	2.104	0.787	0.178

Table 3 presents the descriptive analysis of the dichotomous variables in the study, with the following results: In the gender distribution, males (coded as 1) and females (coded as 2) each had a frequency of 51, accounting for 50.0% of the total sample, indicating a balanced gender distribution (chi-square = 0.000, *df* = 1, sig = 1.000). In the experimental results distribution, honest behavior (coded as 0) had a frequency of 67, accounting for 65.7% of the total sample, while dishonest behavior (coded as 1) had a frequency of 35, accounting for 34.3% of the total sample. This indicates that the proportion of honest behavior in the sample was significantly higher than that of dishonest behavior (chi-square = 10.039, *df* = 1, sig = 0.002).

**Table 3:** Descriptive statistical analysis of dichotomous variables.

**Variable**	**Category**	**Frequency**	**Percentage (%)**
Gender	Male (1)	51	50.0
	Female (2)	51	50.0
Results	Honesty (0)	67	65.7
	Dishonesty (1)	35	34.3

Table 4 examines the multicollinearity among the personality trait dimensions, and the results show that the collinearity statistics for each dimension are within acceptable ranges. Specifically, the tolerance for Neuroticism is 0.775, and the variance inflation factor (VIF) is 1.291; the tolerance for Extraversion is 0.831, and the VIF is 1.204; the tolerance for Psychoticism is 0.873, and the VIF is 1.145. According to common criteria (tolerance > 0.1, VIF < 10), the tolerance for all dimensions is >0.1, and the VIF is < 10, indicating that there is no significant multicollinearity issue among the personality trait dimensions. This makes them suitable for subsequent regression analysis or other multivariate statistical modeling.

**Table 4:** Multicollinearity test.

**Model**		**Collinearity statistics**	
		**Tolerance**	**VIF**
1	Neuroticism	0.775	1.291
2	Extraversion	0.831	1.204
3	Psychoticism	0.873	1.145

### Correlation analysis

Table 5 presents the Pearson correlation results between the experimental outcomes, personality dimensions, and demographic variables. Traditional static models reveal that, except for gender (*r* = −0.227, *p* = 0.022), none of the variables show a significant correlation with honest behavior. However, the correlation analysis between honest behavior and Neuroticism suggests a potential association (*p* = 0.096), which may require more sensitive tools to uncover. Therefore, in subsequent research, machine learning algorithms are employed, and SHAP analysis is used to explore interpretability.

**Table 5:** Correlation analysis between experimental results and variables.

**Model**	**Dishonest behavior**	**Gender**	**Neuroticism**	**Psychoticism**	**Extraversion**	**Living regions**	**Living expense levels**
Dishonest behavior	1.000	−0.227^*^	0.166	0.026	−0.042	−0.092	0.098
		0.022	0.096	0.794	0.674	0.357	0.328
Gender	−0.227^*^	1.000	−0.121	−0.056	0.110	0.080	−0.010
	0.022		0.228	0.574	0.270	0.426	0.923
Neuroticism	0.166	−0.121	1.000	0.280^**^	−0.351^**^	0.057	−0.007
	0.096	−0.228		0.004	< 0.001	0.571	0.947
Psychoticism	0.026	−0.056	0.280^**^	1.000	0.109	0.006	−0.007
	0.794	0.574	0.004		0.277	0.953	0.948
Extraversion	−0.042	0.110	−0.351^**^	0.109	1.000	0.060	−0.100
	0.674	0.270	< 0.001	0.277		0.549	0.319
Living regions	−0.092	0.080	0.057	0.006	0.060	1.000	−0.117
	0.357	0.426	0.571	0.953	0.549		0.243
Living expense levels	0.098	−0.010	−0.007	−0.007	−0.100	−0.117	1.000
	0.328	0.923	0.947	0.948	0.319	0.243	

^*^Significant at the 0.05 level (two-tailed). ^**^Significant at the 0.01 level (two-tailed).

### Difference analysis

Table 6 presents the results of the independent samples *t*-test analysis. The contributions of gender, personality traits (Neuroticism, Extraversion, Psychoticism), urban classification, and living expenses to the experimental outcomes reveal that males are more likely to exhibit dishonest behavior than females, and this difference is statistically significant with a moderate effect size.

**Table 6:** The independent samples *t*-test analysis.

**Variable**	***t***	***df***	**Sig(two-tailed)**	**Cohen's *d***
Gender	2.333	100	0.022	0.492
Neuroticism	1.680	100	0.096	3.403
Extraversion	−0.423	100	0.674	2.691
Psychoticism	0.261	100	0.794	1.408
Living regions	−0.982	100	0.328	1.018
Living expense levels	0.926	100	0.357	2.105

### Model construction and evaluation

**Figure 3:**
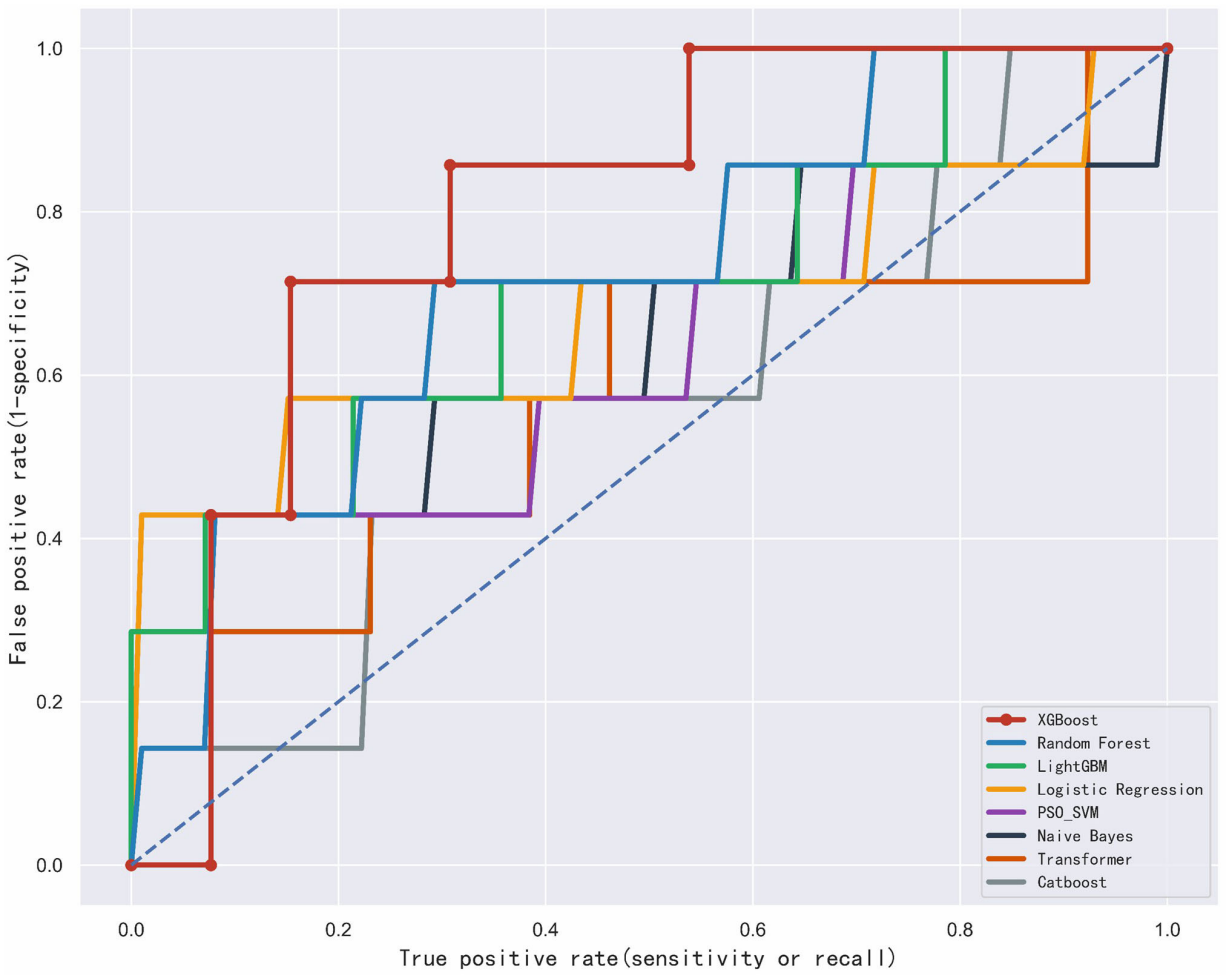
The receiver operating characteristic curve among the four models for dishonest behavior subjects. PSO_SVM, Particle Swarm Optimization Support Vector Machine.

In the training dataset, models including XGBoost, PSO_SVM, Random Forest, Naive Bayes, Logistic Regression, LightGBM, CatBoost, and Transformer were developed. On the test dataset, these models achieved AUC scores of 0.802, 0.725, 0.704, 0.684, 0.637, 0.633, 0.560, and 0.560, respectively (Table 1). The XGBoost model demonstrated the best overall performance and achieved the highest predictive accuracy (AUC = 0.802), while the CatBoost model performed relatively weakly across all metrics, recording the lowest AUC of 0.560, respectively (Table 7 and Figure 3).

**Table 7:** Performance of each model for prediction.

**Model**	**AUC^a^ (%)**	**CI (α = 0.05)**	**Youden's index**	**Specificity**	**Sensitivity (%)**	***F*_1_ score**	**Accuracy (%)**	**PPV^b^**	**NPV^c^**
XGBoost	0.802	0.802 ± 0.074	0.567	0.853	0.714	0.714	0.800	0.714	0.856
Random Forest	0.725	0.725 ± 0.084	0.571	1.000	0.571	0.727	0.857	1.00	0.824
LightGBM	0.704	0.704 ± 0.086	0.354	0.925	0.429	0.545	0.800	0.750	0.765
Logistic Regression	0.684	0.684 ± 0.083	0.198	0.627	0.571	0.500	0.619	0.444	0.750
PSO_SVM^d^	0.637	0.637 ± 0.121	−0.162	0.552	0.286	0.267	0.450	0.250	0.583
Naive Bayes	0.633	0.633 ± 0.087	0.354	0.925	0.429	0.545	0.800	0.750	0.765
Transformer	0.560	0.560 ± 0.088	0.464	0.464	1.000	0.568	0.400	0.368	1.000
Catboost	0.560	0.560 ± 0.093	0.050	0.621	0.429	0.400	0.55	0.375	0.667

^a^AUC, area under the curve.

^b^PPV, positive predictive value.

^c^NPV, negative predictive value.

^d^PSO_SVM, Particle Swarm Optimization Support Vector Machine.

### Explaining the XGBoost model with SHAP method

**Figure 4:**
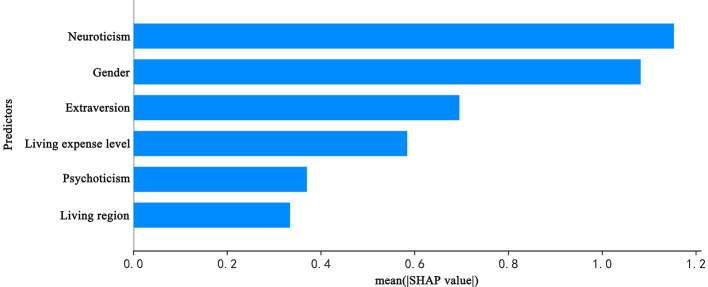
The weights of variables importance.

**Figure 5:**
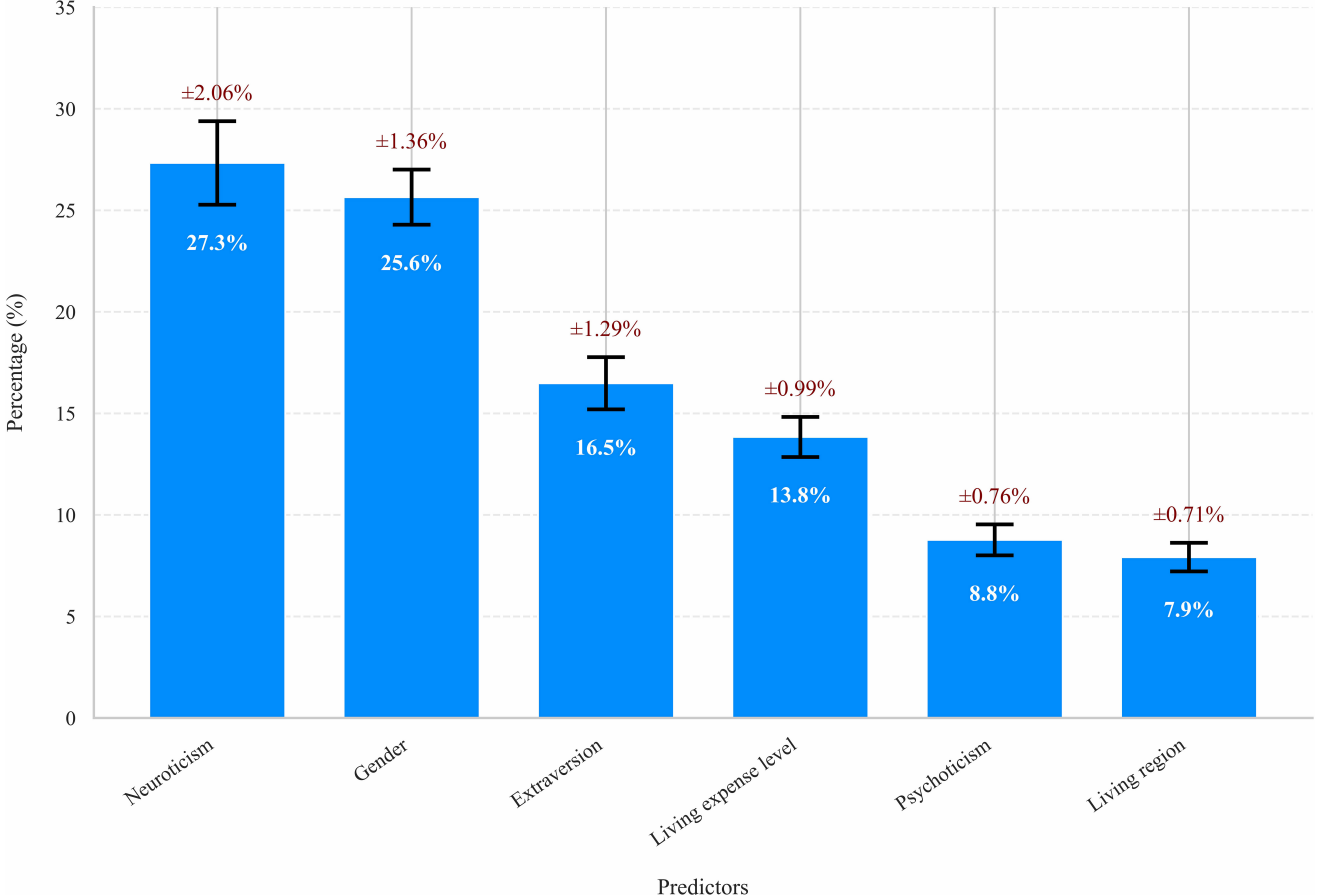
Comparative chart of importance indicator percentages.

The SHAP method was applied to quantify the impact of each predictor variable in the XGBoost model's predictions. The variable importance plot lists the most impactful predictors in descending order (Figure 4). Neuroticism emerged as the most significant predictor, followed by gender, extraversion, living expenses, psychoticism, and city classification. After normalizing the importance metrics in Figure 4 to percentages and adding error bars, the results are presented as Figure 5, enhancing readability.

**Figure 6:**
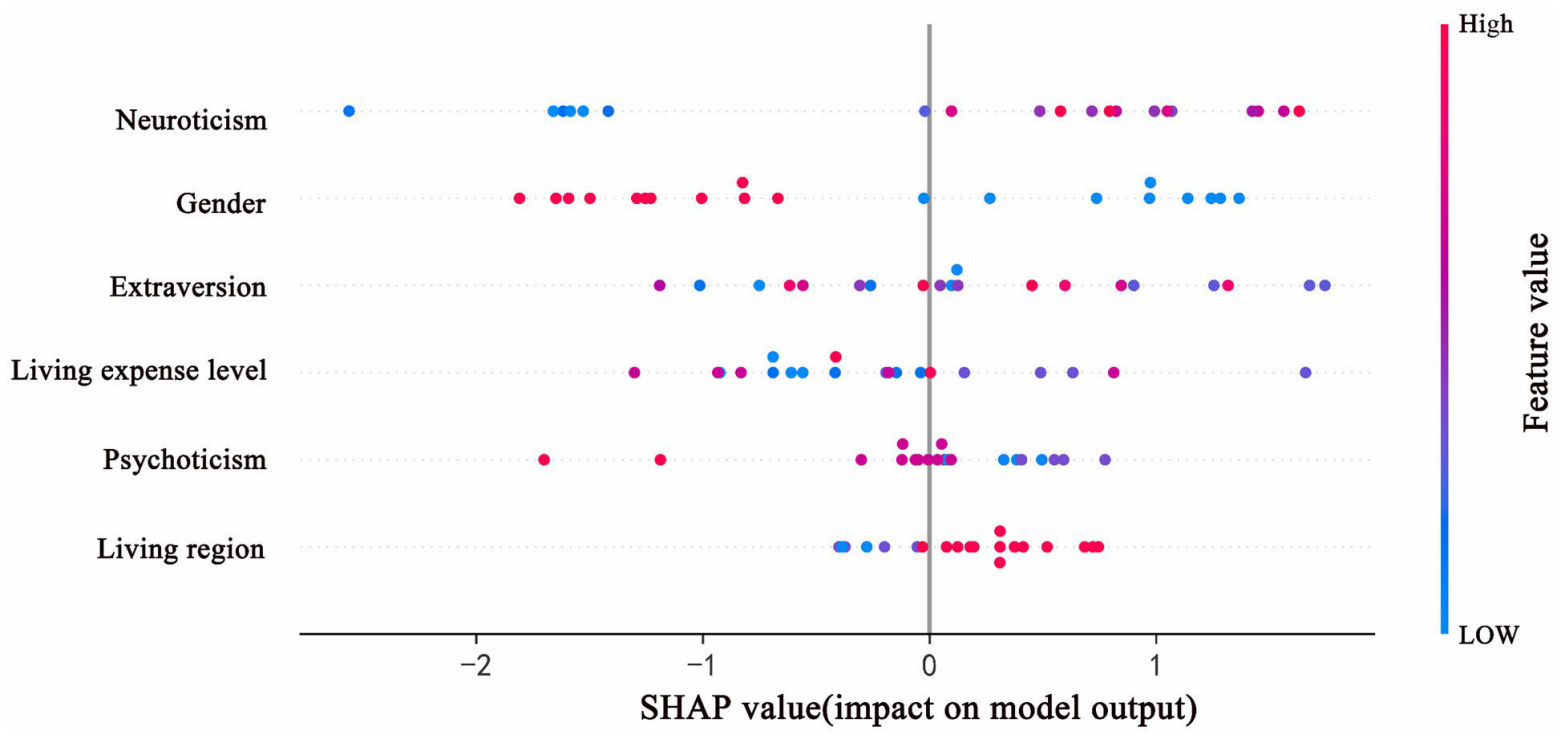
The SHAP values.

Additionally, SHAP values were used to elucidate the positive and negative influences of each predictor on the target outcome (i.e., honesty behavior). As shown in Figure 6, the horizontal placement indicates the association of values with honesty or dishonesty, while the color signals the magnitude of each variable's effect in observations (red for high values, blue for low values). For example, an increase in neuroticism tends to predict dishonest behavior, thereby shifting predictions toward dishonest behavior, whereas being female tends to predict honesty, shifting predictions toward honest behavior.

### SHAP individual force plot

**Figure 7:**
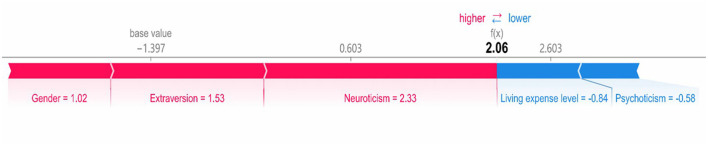
SHAP force plot for a male dishonest subject.

**Figure 8:**
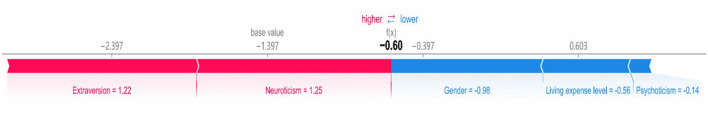
SHAP force plot for a female honest subject.

Figures 7, 8 show individual force plots for two arbitrarily chosen participants, one male and one female. SHAP values determine the predictive features for each participant and quantify each feature's contribution to the prediction of dishonest behavior. Bold values indicate the predicted probability [*f* (*x*)], while the baseline value serves as the model's output in the absence of input data. In these representations, red features on the left signify factors that enhance dishonest behavior, whereas blue features indicate those that decrease it. Arrow length demonstrates each feature's influence on the prediction; the longer the arrow, the greater the impact. Analysis of the individual force plots reveals that the male participant displayed dishonest behavior in the experiment, with gender, neuroticism, and extraversion positively influencing the prediction. In contrast, the female participant demonstrated honest behavior, with gender exerting a negative influence on the prediction outcome.

### SHAP interaction plot for gender and neuroticism

**Figure 9:**
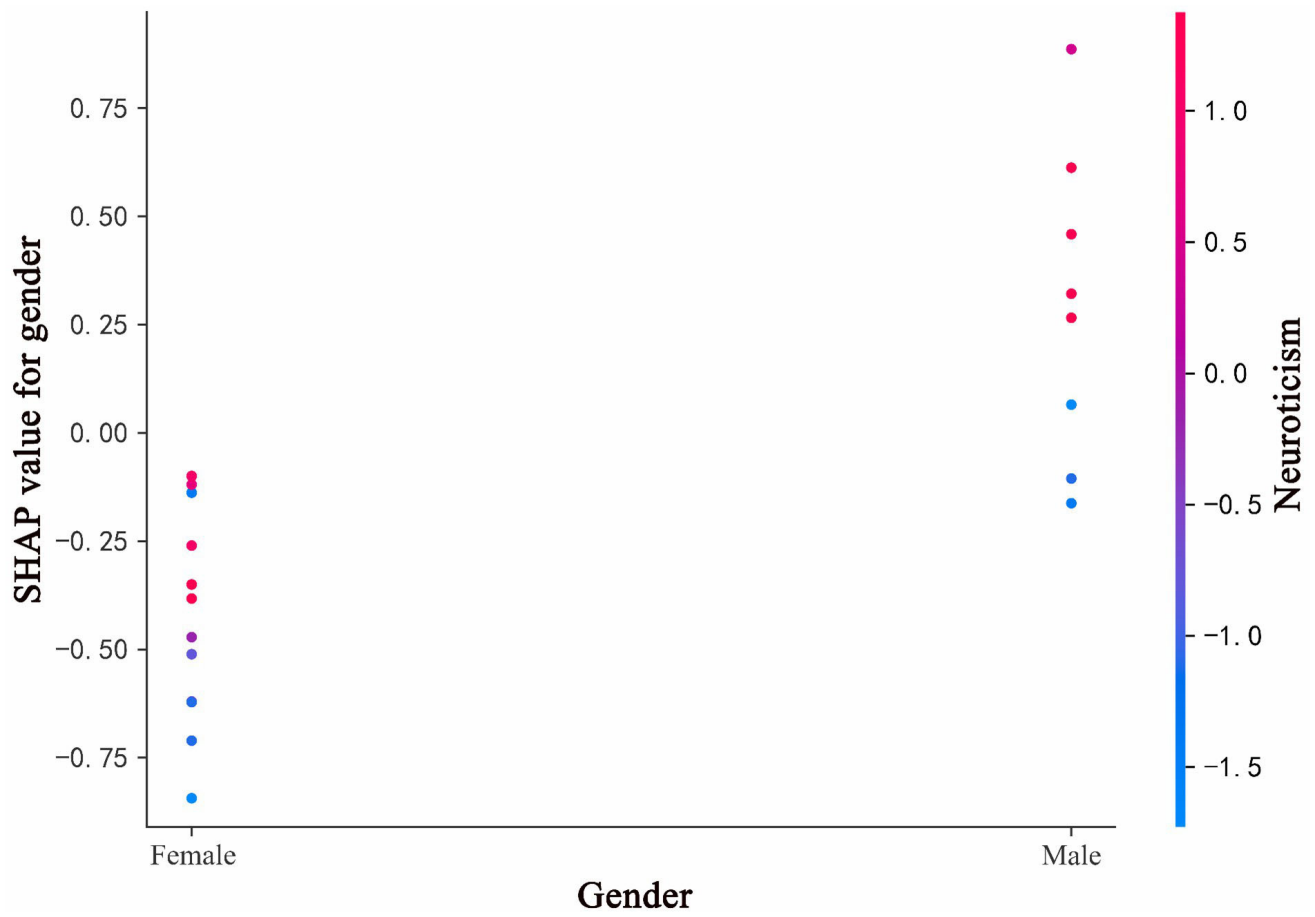
SHAP interaction plot for gender and neuroticism trait.

Figure 9 presents the SHAP dependence plot, illustrating the influence of Gender on the model's predictions and how this influence varies with Neuroticism. Specifically: *X*-axis: Represents the classification of Gender. *Y*-axis: Represents the SHAP values for Gender. SHAP values indicate the contribution of this feature to the model's output. Positive values indicate that the feature increases the predicted probability, while negative values indicate a decrease. Color of Points: The color represents the range of Neuroticism, with blue to red indicating low to high Neuroticism values. Overall Trend: As the Gender value decreases (Male: 1, Female: 2), the SHAP values show an upward trend. This means that, compared to females, male characteristics increase the model's predicted probability of dishonest behavior. When Gender is female, the overall SHAP value is negative, indicating that female characteristics reduce the model's predicted probability of dishonest behavior. Color Distribution: The distribution of points with different colors in the plot reveals the main effect of Neuroticism on Gender. The distribution of blue points (low Neuroticism) and red points (high Neuroticism) across different Gender values helps us understand the relationship between these two features. Detailed Analysis: When Gender is female, the SHAP values are primarily negative, indicating that female characteristics reduce the model's predicted probability of dishonest behavior. The points are distributed with red above and blue below, suggesting that higher Neuroticism values increase the model's predicted probability of dishonest behavior. When Gender is male, the SHAP values are primarily positive, indicating that male characteristics increase the model's predicted probability of dishonest behavior. The points are distributed with red above and blue below, suggesting that higher Neuroticism values further increase the model's predicted probability of dishonest behavior.

## Discussion

### Main findings

In our study, which employed a modified coin-flip task to assess individual honesty behaviors, we developed and validated eight machine learning algorithms to predict honesty based on Eysenck's personality dimensions and gender. Evaluations of the models demonstrated that the XGBoost model surpassed Random Forest, LightGBM, Logistic Regression, PSO-SVM, Naive Bayes, Transformer, and Catboost. Using SHAP to interpret the XGBoost model ensured both high performance and interpretability. SHAP analysis facilitates the allocation of each feature's contribution to the prediction outcome, revealing the direction (positive or negative) and magnitude of each variable's influence on honesty behavior. This method not only enhances the interpretability of the model's results but also quantifies the importance of individual personality traits in the prediction, providing both precision and visual clarity in the explanation.

Our study partially validated the proposed hypotheses: (1) Neuroticism increases the likelihood of dishonest behavior. (2) Males are more likely to exhibit dishonest behavior than females. (3) Gender and personality traits jointly influence dishonest behavior.

This study shares similarities and differences with previous research. The similarity lies in the significant impact of Neuroticism on dishonest behavior, while the difference is that no significant effect of Psychoticism on dishonest behavior was found.

Existing research indicates that the Neuroticism and Psychoticism dimensions of the Eysenck personality model exhibit distinct mechanisms in predicting dishonest behavior. A meta-analysis by Lee et al. (2019) confirmed that psychopathic traits and impulsive behavioral tendencies jointly constitute core predictors of dishonest behavior, with their mechanisms likely rooted in individual differences in emotion regulation and behavioral control systems. The Neuroticism dimension drives morally deviant behavior through the pathway of emotional instability: individuals with high Neuroticism, due to deficits in emotion regulation, are prone to negative emotional states such as anxiety and depression. Such emotional arousal may prompt them to resort to dishonest means (e.g., deception or rule-breaking) for immediate stress relief (Peters et al., 2020). In contrast, the Psychoticism dimension primarily influences behavioral decision-making through deficits in behavioral inhibition, manifesting as a tendency for individuals with high Psychoticism scores to adopt aggressive strategies (Barnes and Malamuth, 1998) or externalized coping mechanisms (e.g., systematic lying; Dodaj et al., 2021) in response to external challenges.

In this study, SHAP analysis indicated that higher neuroticism markedly increased the likelihood of dishonest behavior, and men were more likely than women to engage in dishonest actions—findings corroborated by previous research (Giluk and Postlethwaite, 2015; Huang and Hung, 2013; Kennedy and Kray, 2022; Muñoz García et al., 2021; Pierce and Balasubramanian, 2015; Zhang et al., 2022). Neuroticism exhibited a strong positive predictive association with dishonest behavior, which could be particularly pertinent in the high-stress, complex environments faced by pilots and air traffic controllers. In such contexts, individuals with high neuroticism may be more susceptible to emotional fluctuations, increasing their likelihood of engaging in dishonest behavior under pressure or to gain advantage.

At least one reason for such a relation of neuroticism with dishonest behavior is poor strategies of emotional regulation. One study conducted on creativity and dishonest behavior found that the volatile emotional reaction of the neurotic individuals affects their honesty in the processes of moral decision-making (Zhang et al., 2022). Meta-analysis also pointed out the fact that, even though the relationship between neuroticism and academic dishonest behavior is rather small, neuroticism might exert its indirect influence on dishonest behavior through the influence on emotional and behavioral control (Giluk and Postlethwaite, 2015). Neuroticism is also relatively similar to emotional control. An example is that according to the study of brain imaging research, those people with neuroticism at high-levels have less activity or activity in some of those brain regions like pre-frontal cortex when regulating these negative emotions and even reduced connectivity between the amygdala and pre-frontal cortex when doing such regulatory action. Thus, by this, it might also be estimated that the functionality of neurotic people's control over negative emotions lies in deficits (Yang et al., 2020). This makes them typically acquire inefficient strategies of emotional regulation; hence, they end up developing emotional dysregulation, sitting longer in negative emotional states (Paulus et al., 2016), which may affect dishonest behavior.

However, Psychoticism did not show a significant effect on dishonest behavior, which may be attributed to sample characteristics [due to CAAC requirements, the civil aviation personnel population might have screened out individuals with high Psychoticism traits (Civil Aviation Administration of China [CAAC], 2020, 2024)].

In the experiment, male participants exhibited rule-violation behavior more often than female subjects. According to earlier literature, this could be because more “rule-following” restrictive factors are not imposed during the socialization process upon males, while females are required to be more rule-conforming and obedient to various social norms and rules during the socialization process (Ward and Beck, 1990), which makes male subjects more apt to violate the rules during the feedback stage of the study. Moreover, research cites that men use deceptive ways of getting more rewards than women when the incentive is monetary (Muñoz García et al., 2021). However, during the experiment, some of the male subjects were honest, and that may be due to their habitual honesty or due to gender stereotypes. Research says, for example, dishonestness may be curbed by untrustworthy men in specific social settings due to not wanting to attract negative designations (Schniter and Shields, 2020).

The hypothesis that Eysenck's personality traits and gender predict honesty behaviors is very well-supported by the findings of this research. The predictive model constructed in this research provides practical benefit to selecting and screening civil aviation trainees. The airlines will be better equipped at identifying potential honesty risks in individuals when honesty tendencies are assessed during the selection of personnel for key positions. Besides, the model's application should be expanded for periodic evaluation of active pilots and air traffic controllers to help airlines establish mechanisms of honesty assurance in personnel management that will contribute to the overall enhancement of flight safety. As flight cadets and ATC cadets represent the initial training stage for aviation professionals, this study focuses on this group to investigate the relationship between personality traits and honest behavior. This design aligns with the long-standing emphasis on psychological assessment in talent selection by the Civil Aviation Administration of China (CAAC) and the Civil Aviation Flight University of China (CAFUC; Civil Aviation Administration of China [CAAC], 2012, 2015, 2020).

The findings of this study suggest that the selection process for aviation personnel should pay special attention to candidates' emotional regulation abilities, as individuals with lower Neuroticism scores exhibit a higher tendency toward honest behavior. This trait significantly enhances their adherence to standard operating procedures (SOPs) during training and flight operations, as well as their willingness to proactively report flight safety incidents and psychological issues, thereby effectively improving civil aviation safety and ensuring public travel security. In the fields of pilot training and professional ethics development, it is recommended to tailor Crew Resource Management (CRM) courses for individuals with higher Neuroticism scores, focusing on strengthening communication skills, conflict resolution abilities, team collaboration willingness, and emotional management efficacy. Additionally, periodic psychological counseling can be implemented for high-risk groups based on cumulative risk characteristics of illicit profit, systematically enhancing the industry's overall ethical standards. The machine learning prediction model constructed in this study provides a scientific decision-making framework for aviation human resource management. Leveraging the XGBoost model's in-depth analysis of Eysenck personality traits and gender variables, managers can accurately identify individuals with potential honest risks, particularly male groups with Neuroticism scores exceeding the threshold. SHAP interpretability analysis reveals key influencing factors, enabling the targeted development of emotional regulation training modules and the establishment of gender-differentiated moral incentive mechanisms. The model's predictive results can be integrated into the existing psychological assessment systems of CAAC, CAFUC, and airlines, enabling real-time risk warnings and dynamic evaluation of job suitability. At the level of safety culture construction, intervention strategies based on feature weights can be transformed into scenario-based simulation training systems, dynamically testing honest decision-making thresholds and reinforcing ethical behavior to build preventive ethical intervention mechanisms. This data-driven decision-making system not only overcomes the static analysis limitations of traditional psychological assessments but also captures the non-linear relationships between personality traits and behavior through machine learning, providing an innovative technological pathway for constructing an explainable and actionable aviation safety governance system.

### Limitations

This study encountered several limitations. First, the small sample size affects the stability of the machine learning algorithms. Due to the impact of the pandemic on the civil aviation system, the demand for airlines to train their own flight cadets has significantly decreased, leading to a sharp reduction in the number of cadets sent for training. Additionally, changes in school policies have resulted in a substantial decline in the number of flight cadets who return to school after passing the Private Pilot License (PAE) and Instrument Rating Exam (IRE) for single-engine and multi-engine aircraft. Moreover, most qualified ATC cadets have been relocated to new training facilities, making it difficult to further expand the sample size of flight cadets and ATC cadets on campus. Second, this study employed a laboratory experiment, which has limited external validity. Real-world environments often involve more factors that influence the honest behavior of flight cadets and ATC cadets, and the laboratory setting has inherent limitations in simulating the real-world ethical challenges they face during training. Furthermore, all participants were recruited from the Civil Aviation Flight University of China, and the single-institution participant pool may lead to a lack of diversity in the sample, raising concerns about generalizability. Aviation students may be more inclined to take risks, making it difficult to extend the findings to broader populations. Additionally, participants from a single institution may share similar educational backgrounds, values, or behavioral patterns, resulting in homogeneity in experimental conditions and further reducing external validity. Lastly, although the experiment included a *post-hoc* correction phase to exclude erroneous choices made by participants, the definition of honest behavior was based on the high ethical standards required in their respective professions. However, when applied to a general sample, this definition of honest behavior might be considered overly stringent.

We acknowledge the limitations in the current study's sample size. In future research, we plan to address this by recruiting a larger and more diverse sample, extending across multiple institutions, airlines, and different stages of flight and air traffic control training to enhance external validity. Additionally, future studies could explore alternative methods, such as transforming honest behavior into continuous data, to improve the predictive performance of the models.

## Conclusions

We formulated an interpretable XGBoost predictive model using SHAP to effectively predict individual honesty behaviors based on Eysenck's personality traits and gender. Results identified neuroticism and gender are key predictors, with individuals scoring high in neuroticism—especially males—being more likely to partake in dishonest behavior. This model exhibits potential for application in personnel screening within high-risk industries such as aviation, contributing to improved safety outcomes.

## Data Availability

The raw data supporting the conclusions of this article will be made available by the authors, without undue reservation.

## References

[B1] Abood N. (2019). Big Five traits: a critical review. Gadjah Mada Int. J. Business 21, 159–186. 10.22146/gamaijb.34931

[B2] Ashton M. C. Lee K. (2020). Objections to the HEXACO model of personality structure—and why those objections fail. Euro. J. Pers. 34, 492–510. 10.1002/per.2242

[B3] Ashton M. C. Lee K. De Vries R. E. (2014). The HEXACO honesty-humility, agreeableness, and emotionality factors: a review of research and theory. Pers. Soc. Psychol. Rev. 18, 139–152. 10.1177/1088868314523838 24577101

[B4] Barnes G. E. Malamuth N. M. (1998). Eysenck's theory of personality and sexuality. Psihologija 3, 239–248. 10.1016/0191-8869(84)90048-5

[B5] Bellucci G. Molter F. Park S. Q. (2019). Neural representations of honesty predict future trust behavior. Nat. Commun. 10:5184. 10.1038/s41467-019-13261-8 31729396 PMC6858375

[B6] Bellucci G. Park S. Q. (2020). Honesty biases trustworthiness impressions. J. Exp. Psychol. General 149:1567. 10.1037/xge0000730 31916837

[B7] Bijoy M. H. I. Hasan M. Rahman M. M. Bitto A. K. Rabbani M. Rubi M. A. (2022). “Classifying gender based on life partner choosing factor using supervised machine learning,” in 2022 13th International Conference on Computing Communication and Networking Technologies (ICCCNT) (Kharagpur: IEEE), 1–5. 10.1109/ICCCNT54827.2022.9984537

[B8] Boyle G. J. Stankov L. Martin N. G. Petrides K. V. Eysenck M. W. Ortet G. (2016). Hans J. Eysenck and Raymond B. Cattell on intelligence and personality. Pers. Individ. Differ. 103, 40–47. 10.1016/j.paid.2016.04.029

[B9] Ceken S. Unsal P. (2023). Cabin crew members' silence: a qualitative study with cabin attendants. Int. J. Aviat. Aeronaut. Aerospace 10:6. 10.58940/2374-6793.1827

[B10] Civil Aviation Administration of China [CAAC]. (2012). Regulations on Training and Management of Multi-Crew Pilot Licenses. Available online at: https://www.caac.gov.cn/XXGK/XXGK/GFXWJ/201511/t20151102_8172.html (accessed August 7, 2024).

[B11] Civil Aviation Administration of China [CAAC]. (2015). Pilot Mental Health Guidelines. Available online at: https://www.caac.gov.cn/XXGK/XXGK/GFXWJ/201706/t20170601_44444.html# (accessed August 8, 2024).

[B12] Civil Aviation Administration of China [CAAC]. (2017). The Civil Aviation Administration of China issued the ‘Civil Aviation Industry Credit Management Measures (Trial)'. Available online at: https://www.gov.cn/xinwen/2017-11/14/content_5239534.htm (accessed August 8, 2024).

[B13] Civil Aviation Administration of China [CAAC]. (2020). Release | Implementation Roadmap for the Construction of a Lifecycle Management System for Skills of Civil Aviation Transport Pilots in China. Available online at: http://www.caac.gov.cn/XXGK/XXGK/ZCFB/202012/t20201225_205785.html (accessed August 11, 2024).

[B14] Civil Aviation Administration of China [CAAC]. (2024). Guidelines for Promoting Mental Health of Pilots. Available online at: https://www.caac.gov.cn/XXGK/XXGK/GFXWJ/202410/t20241010_225577.html# (accessed August 12, 2024).

[B15] Cohn A. Maréchal M. A. Tannenbaum D. Zünd C. L. (2019). Civic honesty around the globe. Science 365, 70–73. 10.1126/science.aau8712 31221770

[B16] Dodaj A. Sesar K. Šimić N. (2021). Coping with bullying behavior: the role of Eysenck's personality dimensions and arousability trait. J. Psychol. 9, 63–73. 10.15640/jpbs.v9n2a6

[B17] Dunne R. Matthews O. Vega J. Harper S. Morris T. (2023). Computational methods for predicting human behaviour in smart environments. J. Ambient Intell. Smart Environ. 15, 179–205. 10.3233/AIS-210384

[B18] Eagly A. H. (2013). Sex Differences in Social Behavior: A Social-Role Interpretation. New York, NY: Psychology Press. 10.4324/9780203781906

[B19] Eysenck M. W. (2016). Hans Eysenck: a research evaluation. Pers. Individ. Differ. 103, 209–219. 10.1016/j.paid.2016.04.039

[B20] Gill C. H. D. Berezina E. (2019). Modeling personality structure using semantic relationships: is the heXaco honesty-humility a distinct trait?. Psychol. Russia State Art 12, 89–103. 10.11621/pir.2019.0107

[B21] Giluk T. L. Postlethwaite B. E. (2015). Big Five personality and academic dishonesty: a meta-analytic review. Pers. Individ. Differ. 72, 59–67. 10.1016/j.paid.2014.08.027

[B22] Gomes H. S. Farrington D. P. Defoe I. N. Maia Â. (2022). Field experiments on dishonesty and stealing: what have we learned in the last 40 years? J. Exp. Criminol. 18, 607–637. 10.1007/s11292-021-09459-w

[B23] Greene J. D. Paxton J. M. (2009). Patterns of neural activity associated with honest and dishonest moral decisions. Proc. Natl. Acad. Sci. U.S.A. 106, 12506–12511. 10.1073/pnas.0900152106 19622733 PMC2718383

[B24] Grosch K. Müller S. Rau H. A. Wasserka-Zhurakhovska L. (2022). Selection into Leadership and Dishonest Behavior of Leaders: A Gender Experiment (IHS Working Paper, 19). Wien: Institut für Höhere Studien (IHS). Available online at: https://nbn-resolving.org/urn:nbn:de:0168-ssoar-85105-7

[B25] Guo X. Yin L. (2024). Behavioral dishonesty in multiscenes: associations with trait honesty and neural patterns during (dis) honesty video-watching. Hum. Brain Mapp. 45:e26710. 10.1002/hbm.26710 38853713 PMC11163231

[B26] Gupta V. K. Mortal S. Chakrabarty B. Guo X. Turban D. B. (2020). CFO gender and financial statement irregularities. Acad. Manage. J. 63, 802–831. 10.5465/amj.2017.0713

[B27] Harenski C. L. Antonenko O. Shane M. S. Kiehl K. A. (2008). Gender differences in neural mechanisms underlying moral sensitivity. Soc. Cogn. Affect. Neurosci. 3, 313–321. 10.1093/scan/nsn026 19015084 PMC2607058

[B28] He Y. Gong R. (2010). “A review of integrity tests in personnel selection,” in 2010 International Conference on E-Product E-Service and E-Entertainment (Henan: IEEE), 1–6. 10.1109/ICEEE.2010.5660850

[B29] Hodson G. Book A. Visser B. A. Volk A. A. Ashton M. C. Lee K. (2018). Is the dark triad common factor distinct from low honesty-humility?. J. Res. Pers. 73, 123–129. 10.1016/j.jrp.2017.11.012

[B30] Houdek P. Bahník Š. Hudík M. Vranka M. (2021). Selection effects on dishonest behavior. Judgment Decision Making 16, 238–266. 10.1017/S1930297500008561

[B31] Howard M. C. Van Zandt E. C. (2020). The discriminant validity of honesty-humility: a meta-analysis of the HEXACO, Big Five, and Dark Triad. J. Res. Pers. 87:103982. 10.1016/j.jrp.2020.103982

[B32] Huang H. J. Hung Y. (2013). Gender differences and behavioral integrity: from a social contract perspective. J. Manage. Org. 19, 86–100. 10.1017/jmo.2013.6

[B33] Kambham N. K. Stanley K. G. Bell S. (2018). “Predicting personality traits using smartphone sensor data and app usage data,” in 2018 IEEE 9th Annual Information Technology, Electronics and Mobile Communication Conference (IEMCON) (Vancouver, BC: IEEE), 125–132. 10.1109/IEMCON.2018.8614854

[B34] Kennedy J. A. Kray L. J. (2022). Gender similarities and differences in dishonesty. Curr. Opin. Psychol. 48:101461. 10.1016/j.copsyc.2022.101461 36116425

[B35] Koglbauer I. (2018). Can you understand and predict how people perform in an aviation environment? [Review of the book Aviation psychology and human factors (2nd ed.), by M. Martinussen & D. R. Hunter]. Aviat. Psychol. Appl. Hum. Factors 8, 124–125. 10.1027/2192-0923/a000148

[B36] Kokkinos C. M. Antoniadou N. Voulgaridou I. (2023). Personality profile differences in academic dishonesty and procrastination among Greek university students: a five factor facet-level latent profile analysis. Pers. Individ. Differ. 214:112337. 10.1016/j.paid.2023.112337

[B37] Kunte A. V. Panicker S. (2019). “Analysis of machine learning algorithms for predicting personality: brief survey and experimentation,” in 2019 Global Conference for Advancement in Technology (GCAT) (Bangalore: IEEE), 1–5. 10.1109/GCAT47503.2019.8978469

[B38] Lázaro F. L. Nogueira R. P. Melicio R. Valério D. Santos L. F. (2024). Human factors as predictor of fatalities in aviation accidents: a neural network analysis. Appl. Sci. 14:640. 10.3390/app14020640

[B39] Lee J. J. Hardin A. E. Parmar B. Gino F. (2019). The interpersonal costs of dishonesty: how dishonest behavior reduces individuals' ability to read others' emotions. J. Exp. Psychol. General 148:1557. 10.1037/xge0000639 31305092

[B40] Levine E. E. Cohen T. R. (2018). You can handle the truth: mispredicting the consequences of honest communication. J. Exp. Psychol. General 147:1400. 10.1037/xge0000488 30148388

[B41] Liu C. Li F. Li L. (2021). “Research on gender prediction for social media user profiling by machine learning method,” in 2021 International Conference on Communications, Information System and Computer Engineering (CISCE) (Beijing: IEEE), 831–836. 10.1109/CISCE52179.2021.9445922

[B42] Lundberg S. M. Lee S. I. (2017). A unified approach to interpreting model predictions. Adv. Neural Inf. Process. Syst. 30, 4765–4774. 10.5555/3295222.3295230

[B43] Malesky A. Grist C. Poovey K. Dennis N. (2022). The effects of peer influence, honor codes, and personality traits on cheating behavior in a university setting. Ethics Behav. 32, 12–21. 10.1080/10508422.2020.1869006

[B44] Marcus B. Lee K. Ashton M. C. (2007). Personality dimensions explaining relationships between integrity tests and counterproductive behavior: Big Five, or one in addition?. Pers. Psychol. 60, 1–34. 10.1111/j.1744-6570.2007.00063.x

[B45] Matthews G. (2016). Traits, cognitive processes and adaptation: an elegy for Hans Eysenck's personality theory. Pers. Individ. Differ. 103, 61–67. 10.1016/j.paid.2016.04.037

[B46] McCrae R. R. Costa Jr. P. T. (2021). Understanding persons: from Stern's personalistics to Five-factor theory. Pers. Individ. Differ. 169:109816. 10.1016/j.paid.2020.109816

[B47] McMurtrie K. (2020). Influences on flight crew reporting behaviour: trust and fear of reprisal (Doctoral dissertation). Sydney: UNSW Sydney.

[B48] McMurtrie K. J. Molesworth B. R. (2018). Australian flight crews' trust in voluntary reporting systems and just culture policies. Aviat. Psychol. Appl. Hum. Fact. 8, 11–21. 10.1027/2192-0923/a000131

[B49] Muñoz García A. Gil-Gómez de Liaño B. Pascual-Ezama D. (2021). Gender differences in individual dishonesty profiles. Front. Psychol. 12:728115. 10.3389/fpsyg.2021.728115 34955957 PMC8703141

[B50] Naik H. Dedhia S. Dubbewar A. Joshi M. Patil V. (2022). “Myers briggs type indicator (mbti)-personality prediction using deep learning,” in 2022 2nd Asian Conference on Innovation in Technology (ASIANCON) (Ravet: IEEE), 1–6. 10.1109/ASIANCON55314.2022.9909077

[B51] Nan B. (2020). More than 30% of current Pakistani civil aviation pilots hold fake licenses, says aviation minister. The Paper. Available online at: https://www.thepaper.cn/newsDetail_forward_7998485

[B52] Padrell M. Riba D. Úbeda Y. Amici F. Llorente M. (2020). Personality, cognition and behavior in chimpanzees: a new approach based on Eysenck's model. PeerJ 8:e9707. 10.7717/peerj.9707 32874782 PMC7439959

[B53] Paleczek D. Bergner S. Rybnicek R. (2018). Predicting career success: is the dark side of personality worth considering?. J. Manager. Psychol. 33, 437–456. 10.1108/JMP-11-2017-0402

[B54] Pasha T. Stokes P. R. (2018). Reflecting on the Germanwings disaster: a systematic review of depression and suicide in commercial airline pilots. Front. Psychiatry 9:86. 10.3389/fpsyt.2018.00086 29615937 PMC5869314

[B55] Paulus D. J. Vanwoerden S. Norton P. J. Sharp C. (2016). Emotion dysregulation, psychological inflexibility, and shame as explanatory factors between neuroticism and depression. J. Affect. Disord. 190, 376–385. 10.1016/j.jad.2015.10.014 26546773

[B56] Peters E. M. Bowen R. Balbuena L. (2020). Mood instability and trait anxiety as distinct components of Eysenckian neuroticism with differential relations to impulsivity and risk taking. J. Pers. Assess. 102, 337–347. 10.1080/00223891.2019.1569528 30907661

[B57] Pierce L. Balasubramanian P. (2015). Behavioral field evidence on psychological and social factors in dishonesty and misconduct. Curr. Opin. Psychol. 6, 70–76. 10.1016/j.copsyc.2015.04.002

[B58] Qian M. Wu G. Zhu R. Zhang S. (2000). Development of the revised Eysenck personality questionnaire short scale for Chinese (EPQ-RSC). Acta Psychol. Sin. 32:317.

[B59] Rahm-Knigge R. L. Prince M. A. Conner B. T. (2018). Social interaction anxiety and personality traits predicting engagement in health risk sexual behaviors. J. Anxiety Disord. 57, 57–65. 10.1016/j.janxdis.2018.05.002 29759915

[B60] Revelle W. (2016). Hans Eysenck: personality theorist. Pers. Individ. Differ. 103, 32–39. 10.1016/j.paid.2016.04.007

[B61] Riggio R. E. Salinas C. Tucker J. (1988). Personality and deception ability. Pers. Individ. Differ. 9, 189–191. 10.1016/0191-8869(88)90050-5

[B62] Salima M. M'hammed S. Messaadia M. Benslimane S. M. (2023). “Machine learning for predicting personality traits from eye tracking,” in 2023 International Conference on Decision Aid Sciences and Applications (DASA) (Annaba: IEEE), 126–130. 10.1109/DASA59624.2023.10286789

[B63] Schniter E. Shields T. W. (2020). Gender, stereotypes, and trust in communication. Hum. Nat. 31, 296–321. 10.1007/s12110-020-09376-3 32915411

[B64] Scigała K. A. Schild C. Heck D. W. Zettler I. (2019). Who deals with the devil? Interdependence, personality, and corrupted collaboration. Soc. Psychol. Pers. Sci. 10, 1019–1027. 10.1177/1948550618813419

[B65] Sen A. Akin A. Canfield D. V. Chaturvedi A. K. (2007). Medical histories of 61 aviation accident pilots with postmortem SSRI antidepressant residues. Aviat. Space Environ. Med. 78, 1055–1059. 10.3357/ASEM.2030.2007 18018438

[B66] Song X. (2022). “Prediction of people's abnormal behaviors based on machine learning algorithms,” in 2022 International Conference on Machine Learning and Intelligent Systems Engineering (MLISE) (Guangzhou: IEEE), 406–409. 10.1109/MLISE57402.2022.00087

[B67] Speer S. P. Smidts A. Boksem M. A. (2020). Individual differences in (dis) honesty are represented in the brain's functional connectivity: robust out-of-sample prediction of cheating behavior. bioRxiv 2020–05. 10.1101/2020.05.12.091116 34861396

[B68] Sulitzeanu-Kenan R. Tepe M. Yair O. (2022). Public-sector honesty and corruption: field evidence from 40 countries. J. Public Admin. Res. Theory 32, 310–325. 10.1093/jopart/muab033

[B69] Tangney J. P. Stuewig J. Mashek D. J. (2007). Moral emotions and moral behavior. Annu. Rev. Psychol. 58, 345–372. 10.1146/annurev.psych.56.091103.070145 16953797 PMC3083636

[B70] Tobacyk J. J. (2004). A revised paranormal belief scale. Int. J. Transpers. Stud. 23:11. 10.24972/ijts.2004.23.1.94

[B71] U.S. Department of Justice (2021). Former Boeing 737 MAX Chief Technical Pilot Indicted for Fraud. Available online at: https://www.justice.gov/opa/pr/former-boeing-737-max-chief-technical-pilot-indicted-fraud (accessed August 15, 2024).

[B72] Ward D. A. Beck W. L. (1990). Gender and dishonesty. J. Soc. Psychol. 130, 333–339. 10.1080/00224545.1990.9924589

[B73] Xiong S. Xu Y. Zhang B. Zhu L. Xie J. (2022). Smartphone addiction and Eysenck's personality traits among Chinese adolescents: a meta-analysis. Front. Psychol. 12:794112. 10.3389/fpsyg.2021.794112 35185692 PMC8854182

[B74] Yan N. Au O. T. S. (2019). Online learning behavior analysis based on machine learning. Asian Assoc. Open Univ. J. 14, 97–106. 10.1108/AAOUJ-08-2019-0029

[B75] Yang J. Mao Y. Niu Y. Wei D. Wang X. Qiu J. (2020). Individual differences in neuroticism personality trait in emotion regulation. J. Affect. Disord. 265, 468–474. 10.1016/j.jad.2020.01.086 32090774

[B76] Yang Q. Zhang W. Liu S. Gong W. Han Y. Lu J.. (2023). Unraveling controversies over civic honesty measurement: an extended field replication in China. Proc. Natl. Acad. Sci. U.S.A. 120:e2213824120. 10.1073/pnas.2213824120 37428923 PMC10629568

[B77] Zaobao L. (2024). Cathay Pacific Trainee Pilots Involved in Frequent Accidents Banned from Solo Flights by U.S. Training Center. Available online at: https://www.zaobao.com/realtime/china/story20240606–3836954 (accessed August 10, 2024).

[B78] Zhang C. Wu J. Yang Z. Perceval G. (2022). How does creativity influence dishonest behavior? An empirical study of Chinese students. Ethics Behav. 32, 147–161. 10.1080/10508422.2020.1869552

[B79] Zhang F. Yu G. T. Li Y. J. Jiang L. Dong L. (2007). Individual and organizational factors affecting Chinese Civil aviation safety: event analysis based on HFACS framework. China Saf. Sci. J. 17, 67–74. 10.16265/j.cnki.issn1003-3033.2007.10.006

[B80] Zumma M. T. Munia J. A. Halder D. Rahman M. S. (2022). “Personality prediction from twitter dataset using machine learning,” in 2022 13th International Conference on Computing Communication and Networking Technologies (ICCCNT) (Kharagpur: IEEE), 1–5. 10.1109/ICCCNT54827.2022.9984495

